# Variability in the Correlation between Asian Dust Storms and Chlorophyll *a* Concentration from the North to Equatorial Pacific

**DOI:** 10.1371/journal.pone.0057656

**Published:** 2013-02-27

**Authors:** Sai-Chun Tan, Xiaohong Yao, Hui-Wang Gao, Guang-Yu Shi, Xu Yue

**Affiliations:** 1 State Key Laboratory of Numerical Modeling for Atmospheric Sciences and Geophysical Fluid Dynamics, Institute of Atmospheric Physics, Chinese Academy of Sciences, Beijing, China; 2 Key Laboratory of Marine Environment and Ecology (Ministry of Education of China), Ocean University of China, Qingdao, China; 3 School of Engineering and Applied Sciences, Harvard University, Cambridge, Massachusetts, United States of America; University of Delaware, United States of America

## Abstract

A long-term record of Asian dust storms showed seven high-occurrence-frequency centers in China. The intrusion of Asian dust into the downwind seas, including the China seas, the Sea of Japan, the subarctic North Pacific, the North Pacific subtropical gyre, and the western and eastern Equatorial Pacific, has been shown to add nutrients to ocean ecosystems and enhance their biological activities. To explore the relationship between the transported dust from various sources to the six seas and oceanic biological activities with different nutrient conditions, the correlation between monthly chlorophyll *a* concentration in each sea and monthly dust storm occurrence frequencies reaching the sea during 1997–2007 was examined in this study. No correlations were observed between dust and chlorophyll *a* concentration in the <50 m China seas because atmospheric deposition is commonly believed to exert less impact on coastal seas. Significant correlations existed between dust sources and many sea areas, suggesting a link between dust and chlorophyll *a* concentration in those seas. However, the correlation coefficients were highly variable. In general, the correlation coefficients (0.54–0.63) for the Sea of Japan were highest, except for that between the subarctic Pacific and the Taklimakan Desert, where it was as high as 0.7. For the >50 m China seas and the North Pacific subtropical gyre, the correlation coefficients were in the range 0.32–0.57. The correlation coefficients for the western and eastern Equatorial Pacific were relatively low (<0.36). These correlation coefficients were further interpreted in terms of the geographical distributions of dust sources, the transport pathways, the dust deposition, the nutrient conditions of oceans, and the probability of dust storms reaching the seas.

## Introduction

The deserts in China such as the Taklimakan, Badain Jaran, Tengger, and Ulan Buh Deserts are major sources of Asian dust aerosol. They account for 63% of the total dust loadings of Asian dust storms [1]. When this dust is transported to the ocean, it provides macronutrients (such as nitrogen and phosphorus) and micronutrients (such as iron) to surface ecosystems of the oceans [2]–[5]. Dust particles sometimes react with atmospheric anthropogenic pollutants when they cross over industrial cities, altering the bioavailability of Fe and adding more nutrients such as N and P [6]–[8]. The deposition fluxes of nutrients associated with the intrusion of Asian dust into the marginal seas of the Asian continent and the Pacific Ocean were found to enhance phytoplankton growth [9]–[14]. In high-nutrient low-chlorophyll (HNLC) regions, Asian dust input has been reported to increase biomass (a near doubling) by providing Fe and other micronutrients [9]. Calil *et al*., [12] reported that in low-nutrient low-chlorophyll (LNLC) seas such as the North Pacific subtropical gyre, some algae bloom events in the Fe–P co-limited region were associated with Fe supply via atmospheric dust deposition. Our recent study also showed that spring bloom events in the central southern Yellow Sea, characterized as a mesotrophic water mass, could be triggered by the inputs of N and Fe carried by dust storms [13].

With the input of Asian dust derived from different sources, the responses of chlorophyll *a* concentration among HNLC, LNLC, and the China seas may not be the same. Nutrient conditions in those seas are different, e.g. the concentrations of macronutrients in the China seas are higher than those in the LNLC and lower than those in HNLC waters (Table 1). The nutrient condition determines the type of nutrient limitation on biological activities in each sea. The correlation of dust frequency with chlorophyll in those seas can be highly variable. Comparative studies of the similarities and differences could improve the understanding of the relationship between the chlorophyll *a* concentration and Asian dust, but the studies have been very limited.

**Table 1:** The annual mean nutrients (µmol L^−1^) and chlorophyll *a* concentration (mg m^−3^) in the six study sea areas.

Parameters	China seas	Sea of Japan	SubarcticNorth Pacific	North Pacific subtropical gyre	westernEquatorial Pacific	EasternEquatorial Pacific
Region with a depth deeper than 50 m						
nitrate	0.89 (±0.80)	2.07 (±1.78)	10.97 (±5.28)	0.19 (±0.21)	0.39 (±0.39)	3.63 (±2.56)
phosphate	0.22 (±0.08)	0.31 (±0.13)	1.17 (±0.37)	0.13 (±0.05)	0.18 (±0.08)	0.50 (±0.17)
silicate	6.16 (±1.81)	11.03 (±2.76)	23.18 (±10.22)	2.32 (±1.13)	2.03 (±0.58)	2.93 (±1.31)
chlorophyll *a* concentration	0.48 (±0.47)	0.62 (±0.23)	0.72 (±0.69)	0.07 (±0.04)	0.13 (±0.14)	0.16 (±0.06)
Region with a depth shallower than 50 m						
nitrate	1.50 (±1.58)	2.01 (±1.14)	5.55 (±4.03)	0.05 (±0.01)	0.36 (±0.22)	4.50 (±0)
phosphate	0.41 (±0.11)	0.30 (±0.12)	1.07 (±0.20)	0.14 (±0.02)	0.25 (±0.07)	0.63 (±0)
silicate	8.78 (±3.74)	12.89 (±4.40)	17.69 (±6.06)	1.63 (±0.01)	2.92 (±1.33)	4.19 (±0)

The nutrients data are World Ocean Atlas 2009 and are obtained from National Oceanographic Data Center. Chlorophyll *a* concentration is calculated from SeaWiFS products. The data in the table and the parenthesis are the averages and 1 standard deviation, respectively.

**Figure 1:**
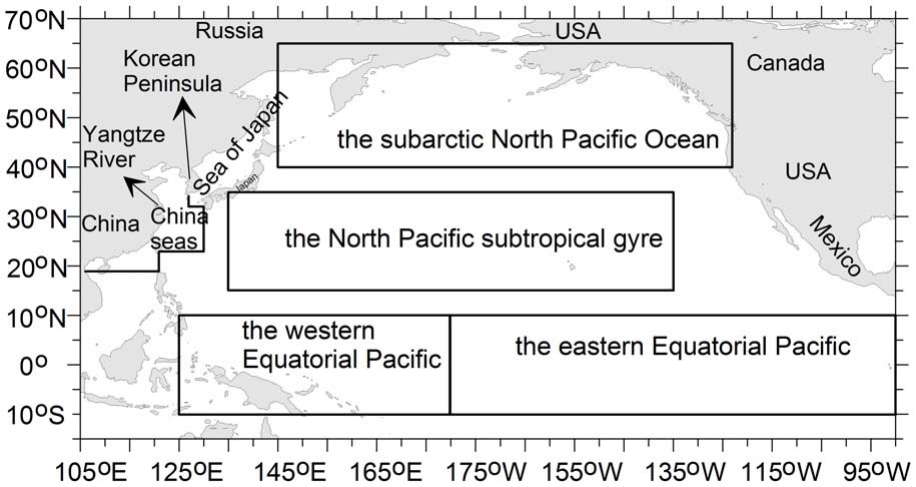
Map of the study sea areas in the Pacific Ocean. The six sea areas are: the China seas, the Sea of Japan, the subarctic North Pacific (40–65°N, 145°E–123°W), the North Pacific subtropical gyre (15–35°N, 135°E–135°W), the western Equatorial Pacific (10°S–10°N, 125–180°E), and the eastern Equatorial Pacific (10°S–10°N, 180°W–90°W).

In this paper, the occurrence frequency and spatial distribution of Asian dust storms from 1997 to 2007 were examined using 1954 to 1996 as reference. A correlation analysis, forward air mass trajectory analysis, and analyses of the dust deposition and the nutrient conditions of the oceans were combined to study the impact of dust storm events occurring in different source zones on chlorophyll *a* concentrations in six areas distributed from marginal seas to the Pacific Ocean (Figure 1).

### Methodology

In this study, the marginal sea and open ocean in the North and Equatorial Pacific were classified into six study areas based on geographical locations and nutrient conditions: 1) the China seas, 2) the Sea of Japan (also known as the East Sea), 3) the subarctic North Pacific Ocean, 4) the North Pacific subtropical gyre, 5) the western Equatorial Pacific Ocean, and 6) the eastern Equatorial Pacific Ocean (Figure 1). Coastal seas with a depth shallower than 50 m were excluded because in these areas, terrestrial processes, such as nutrient input from rivers and/or increased turbidity due to river input with a high content of suspended particulate substances, have dominant impacts on phytoplankton growth. As listed in Table 1, the subarctic North Pacific and the eastern Equatorial Pacific are commonly considered as HNLC regions [7], whereas the North Pacific subtropical gyre is characterized as a LNLC region [12]. The macronutrients and chlorophyll *a* concentration were low in the western Equatorial Pacific (Table 1), and it could be considered as a LNLC region. The China seas and the Sea of Japan are marginal seas of the North Pacific. They were mesotrophic in comparison to the other four seas, and their macronutrient concentrations were lower than that in HNLC waters but higher than that in LNLC waters (Table 1). This definition is also consistent with the classification of biogeochemical provinces for the Pacific Ocean by Longhurst, [15].

The chlorophyll *a* concentration is the SeaWiFS Level-3 standard mapped image products provided by the National Aeronautics and Space Administration, Goddard Space Flight Center, Ocean Biology Processing Group (NASA GSFC OBPG). The spatial resolution of chlorophyll *a* concentration was 9×9 km.

**Figure 2:**
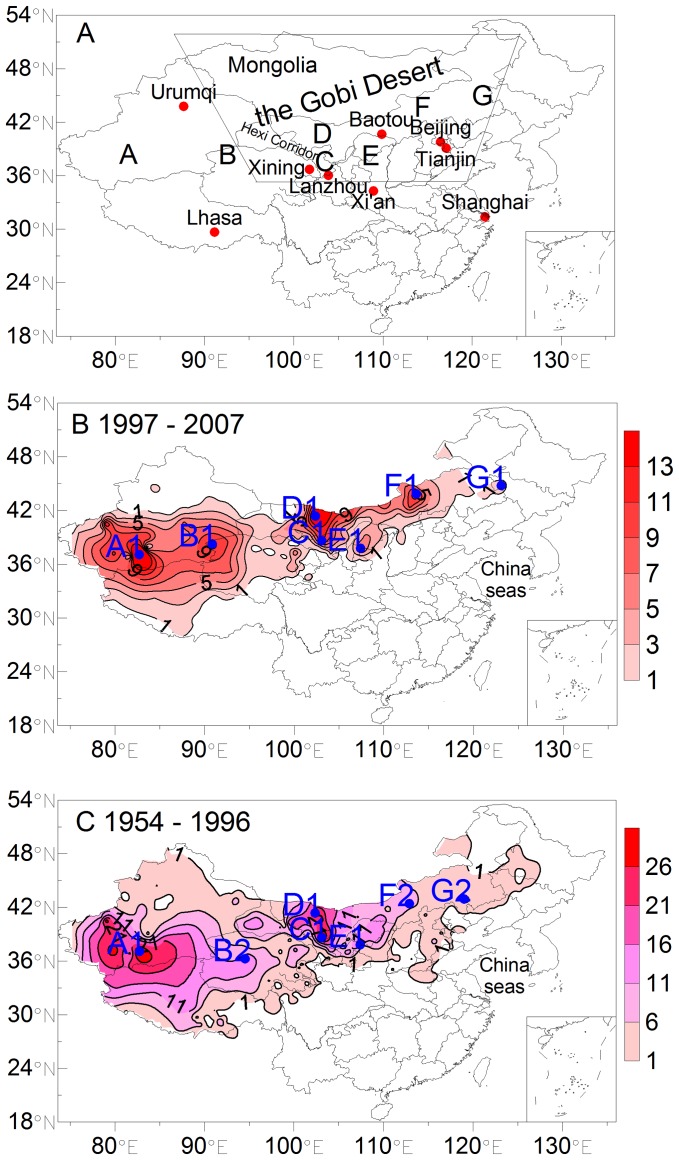
The locations of region A, B, C, D, E, F, G and some cities (A) and the contours of averaged occurrence frequency of dust storms (days per year) in China during 1997–2007 (B) and 1954–1996 (C). (A): A: the Taklimakan Desert, B: the southeastern Xinjiang and Qinghai-Xizang region, C: the Hexi Corridor, D: the west of Inner Mongolia, E: Hetao Region, F: the Hunshandake Desert, G: the Horqin Sandy Land. The frame shows the Gobi Desert. (B) and (C): A1, B1, B2, C1, D1, E1, F1, F2, G1, and G2 are the stations with the highest frequencies of dust storms, and they are Minfeng, Mangya, Golumd, Minqin, Guaizihu, Yanchi, Sunitezuoqi, Zhurihe, Tongyu, and Wenniuteqi, respectively.

The dust storm datasets at 753 meteorological stations in China during 1997–2007 were obtained from the National Meteorological Information Center, China Meteorological Administration (NMIC/CMA). At each station, dust storm weather phenomena were defined as storms with minimum visibility of ≤1 km and instantaneous maximum wind speed of ≥10 m s^−1^ [16]. The datasets includes visibility, wind speed, and the starting and ending time of dust storms on each day at each station. Therefore, the monthly or annual occurrence frequencies of dust storms and the duration of each dust storm can be obtained. Seven dust source zones, shown from west to east in Figure 2A, were labeled as A (the Taklimakan Desert), B (the southeastern Xinjiang and Qinghai-Xizang region including the Qaidam Basin and Kumtag Deserts), C (the Hexi Corridor), D (the west of Inner Mongolia including the Badain Jaran, Ulan Buh, and Tengger Deserts), E (the Hetao Region including the Hobq and Mu Us Deserts and Loess Plateau), F (the Hunshandake Desert), and G (the Horqin Sandy Land). For comparison, the dust storms during 1954–1996 were also characterized using the same dataset.

To examine the impacts of dust storms occurring at different source zones on the six sea areas, a forward trajectory analysis was performed using the Hybrid Single Particle Lagrangian Integrated Trajectory (HYSPLIT) model [17] with inputs from the National Centers for Environmental Prediction/the National Center for Atmospheric Research (NCEP/NCAR) global reanalysis meteorological data. Considering that the lidar measurements and electron microscopy demonstrated that dust layers can be found in the middle to upper free troposphere from the dust source to downwind regions in East Asia [18], the percentage of dust storms from each dust source zone at lower altitudes (3 km in this case) and higher altitudes (7 km in this case) reaching each sea are listed in Table 2. The 3 km and 7 km were the same as Matsuki *et al*., [19]. The percentage was defined as the ratio of the number of trajectories from a dust source zone reaching a sea to the total frequency of dust storms with a duration over 1 hour calculated from the observed starting and ending time during 1997–2007 at the center stations in A, B, C–D–E–F, and G (245, 56, 86–140–65–100, and 20 times, respectively). The trajectories from G are shown in Figure S1 as an example and the trajectories from the other three source zones are similar. To calculate the correlation between dust storm frequencies and chlorophyll *a* concentrations in each sea, the frequencies at A, B, C–D–E–F, and G reaching each sea were used.

**Table 2:** The fraction (percentage) of impacts of dust storms occurring at each dust source zone (A, B, C–D–E–F and G shown in Figure 2A) on the six study sea areas during 1997–2007.

Sea areas	Altitude 3 km				Altitude 7 km			
	A	B	C–D–E–F	G	A	B	C–D–E–F	G
China seas	24	66	51	5	65	57	49	0
Sea of Japan	42	71	74	75	67	84	78	75
subarctic North Pacific	48	84	77	85	62	88	83	85
North Pacific subtropical gyre	23	61	42	15	64	52	56	40
western Equatorial Pacific	0	4	2	5	5	4	4	0
eastern Equatorial Pacific	1	0	1	5	2	0	1	0

The fraction was obtained through forward trajectory analysis from 3 km and 7 km (the altitude of the starting point in the forward trajectory analysis) above the six stations shown in Figure 2B.

The total dust deposition (including dry and wet depositions) data was simulated by the Global Transport Model of Dust (GMOD), which is driven by the meteorological conditions from the Institute of Atmospheric Physics grid-point nine-layer atmospheric general circulation model (IAP9L-AGCM). The simulation is carried out on 5°×4° grids for a 20-year period to estimate the present-day climatology. The simulated dust climatology by GMOD model was validated with both station-based and satellite observations by Yue *et al*., [20] and it reproduces observed dust concentrations, logarithmic total deposition, aerosol size distribution, and aerosol optical thickness reasonably well.

## Results and Discussion

### Overview of Dust Storms Observed in China

The spatial distributions of the annual occurrence frequency (days per year) of Asian dust storms from 1997 to 2007 in China are compared to that from 1954 to 1996 in Figure 2. Six high frequency centers (i.e., A, B, C, D, E, and F) were identified (Figure 2B). Although the occurrence frequency was relatively low at source zone G, these dust events were found to reach regularly the China seas, the Sea of Japan, and the North Pacific through long-range transport [21]. The highest frequencies at A, D, F, B, C, E, and G were 29.4, 18.9, 13.6, 11.8, 11.7, 7.1, and 2.4 days per year from 1997 to 2007, respectively. Using 1954–1996 as a reference, the highest frequencies at A (34.1 days per year) and B (13.7 days per year) decreased by about 14%, and they decreased by about 64%, 23%, 66%, and 71% at C (32.4 days per year), D (24.6 days per year), E (21.1 days per year), and G (8.4 days per year), respectively (Figure 2C). However, the highest frequencies increased by about 81% at F (7.5 days per year). The change in the East Asian atmospheric circulation under global warming, not desertification conditions, could be a major factor in the change in dust storms on decadal timescales [16], [22].

During the periods of 1997–2007 and 1954–1996, the highest frequencies at A, C, D, and E occurred at the same stations. The stations were Minfeng (A1 in Figure 2B and C), Minqin (C1), Guaizihu (D1), and Yanchi (E1). The highest values shifted slightly to the north at B, F, and G from 1954–1996 to 1997–2007. The stations shifted from Golumd, Zhurihe, and Wenniuteqi (B2, F2, and G2 in Figure 2C) to Mangya, Sunitezuoqi, and Tongyu (B1, F1, and G1 in Figure 2B).

### Correlations between Dust Events and Chlorophyll *a* Concentration

**Figure 3:**
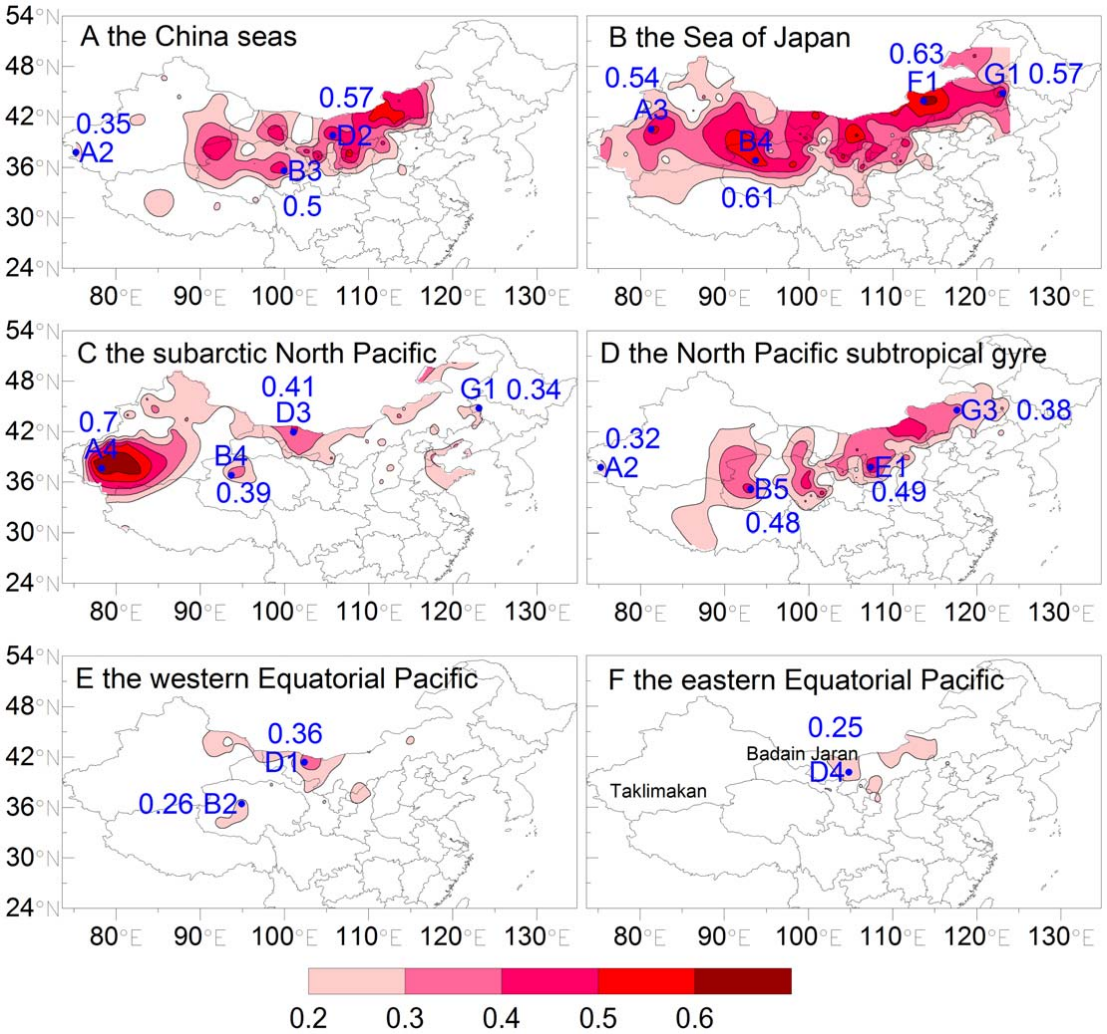
The correlation between monthly chlorophyll *a* concentration (mg m^−3^) in the six sea areas and monthly occurrence frequency of dust storms (days per month) for the period from September 1997 to December 2007. The minimum contour is 0.2 with significance level of 0.05. The stations with the largest correlation coefficient were shown. B2, D1, E1, F1 and G1 are the same stations as Figure 2. A2, A3, A4, B3, B4, B5, D2, D3, D4, and G3 are the stations of Tashkurghan, Alar, Pishan, Xinghai, Xiaozaohuo, Wudaoliang, Jilantai, Ejinaqi, Bayinmaodao, and Xiujimqinqi, respectively.

**Figure 4:**
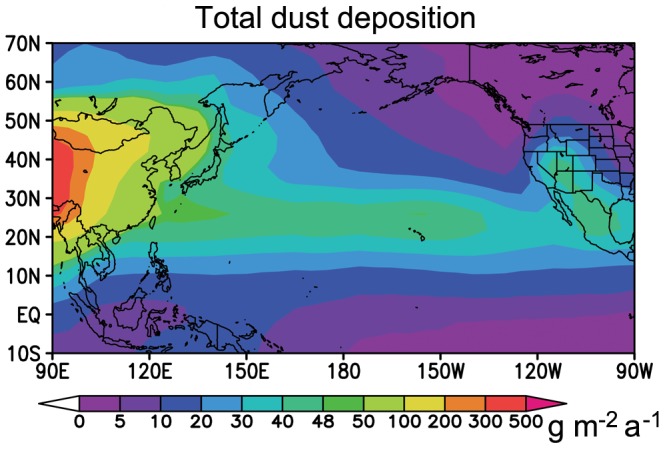
Simulated annual total dust deposition (including dry and wet depositions, unit: g m^−2^ a^−1^).

The four zones (C, D, E, and F) in the Gobi Desert (Figure 2A) are geographically close to one another and therefore were grouped together for correlation analysis. Figure 3 shows the contour distributions of correlation coefficients between monthly chlorophyll *a* concentration (mg m^−3^) in the six study sea areas and monthly occurrence frequency of dust storms (days per month) observed in A, B, C–D–E–F, and G reaching each sea from September 1997 to December 2007 (124 months in total). The significance level is <0.05. The correlation coefficients are discussed below:

1. The highest correlation coefficients between monthly chlorophyll *a* concentration in the >50 m China seas and dust storms at B and C–D–E–F were 0.5 and 0.57 (Figure 3A), respectively, suggesting that these dust events could reach the China seas, as supported by Zhang and Gao, [21], and promote its chlorophyll *a* concentration. Forward trajectories also indicated that most low-altitude dust particles from sources B and C–D–E–F reached the China seas (Table 2). The correlation was relatively low at A in the Taklimakan Desert, which is surrounded by high mountains, except in the east. Only dust particles with sufficient elevation can be transported out of this source region, and these are less likely to enter the marine atmospheric boundary layer and therefore may not affect the biological activities of the China seas [13]. Only 24% of the low-altitude dust particles from A were transported to the China seas (Table 2). The dust particles from source G had less impact on the China seas resulting in no correlation there (Table 2). The ecosystem was P limited in the area close to the Yangtze River whereas N limitation occurred in the other areas of the China seas and it may have been limited by available Fe [23], [24]. According to the Redfield ratio of Si:N:P = 16∶16∶1, the China seas (>50 m) was N limited because the annual averaged Si:N:P is about 28∶4∶1 (Table 1). Dust particles were important carriers of N and Fe, especially when they passed over highly polluted cities, where the NO_2_ and SO_2_ concentrations were high [8]. Research indicated that the reaction of pollutants (e.g., HNO_3_) with Asian dust could produce >70% of the nitrate under heavy dust loadings [25]. Some atmospheric process (e.g., acid processes) can produce more soluble Fe-containing dust [6]. The spring bloom in the China seas could be affected by these nutrients carried by Asian dust, as much of dust (68%) reached the China seas in spring (Table S1) and the spring dust deposition was also the largest (Table 3).
2. Monthly chlorophyll *a* concentration in the Sea of Japan was moderately correlated with the monthly occurrence frequencies of all dust storms, with correlation coefficients ranging from 0.54 to 0.63 (Figure 3B). In fact, these correlations were the highest among all sea areas, and forward trajectories indicated that more than 42% of the dust events were transported from these source zones (Table 2) and the total dust deposition in the Sea of Japan was also the largest among the six seas (Table 3). The percentage reaching the sea in spring amounted to 66% (Table S1) and the spring dust deposition accounted for 46% of the annual one (Table 3). The bioavailable Fe supply from Asian dust has been proposed to potentially result in an earlier initiation of bloom and/or an increase in chlorophyll *a* concentrations in the Sea of Japan in spring [11]. Takata *et al*., [26] reported that high vertically integrated total dissolvable Fe inventories in the Sea of Japan in fall may result from atmospheric Fe input. The calculated percentage of Asian dust reaching the Sea of Japan in fall was 11% (Table S1), indicating that the phytoplankton growth in fall could be also affected by dust particles. The Sea of Japan is generally higher in macronutrient concentration than the China seas (Table 1), so the biological activities of the former should be less sensitive to external nutrient inputs than the latter. The question is why the correlation obtained for the Sea of Japan is higher than that for the China seas. It appears that the nutrient mechanisms reported in the literature cannot alone explain the lower correlation for the China seas. It may be that more dust storms reach and deposit into the Sea of Japan during spring to fall than reach the China seas (Tables 2 and 3, and Table S1). Furthermore, it can be seen from Figures S2 and S3 that some of the Asian dust is transported to the Sea of Japan over less polluted areas (low aerosol optical depth and NO_2_ column density), whereas that arriving at the China seas is transported over polluted areas (high aerosol optical depth and NO_2_ column density) and could pick up toxic metals, potentially decreasing the biological activities [27]. Asian dust may have a positive impact (providing nutrients) and/or a negative one (causing toxicity) on the biological activities of the China seas.
3. The subarctic North Pacific had the highest correlation coefficient with region A (Figure 3C). The largest percentage of dust events in which low-altitude dust particles reached the subarctic North Pacific came from area A, with many high-altitude dust particles reaching the subarctic North Pacific (Table 2). Additionally, eolian Fe inputs to this HNLC water when light was not the limiting factor, due to the fact that most dust storms occurred in April–July at A [15], [16] and the total dust deposition in spring and summer was also very large (Table 3), resulted in the highest correlation between dust frequency and chlorophyll *a* concentration. The subarctic North Pacific also showed a significant correlation with the other three dust sources (B, C–D–E–F, and G), but the correlation coefficient was lower than that for A owing to the fact that most dust storms occurred in February–March or March–May [16], when light may still have limited phytoplankton growth, particularly at higher latitudes [15]. Yuan and Zhang, [10] reported a high correlation coefficient between the monthly KNOT (the Kyodo North Pacific Ocean Time-series station, 44°N,155°E)-derived lithogenic material flux at a depth of 924 m and monthly occurrence of blowing dust events at A (the maximum is about 0.6) and D (the maximum is about 0.5). Their results were comparable to our results of 0.7 and 0.41 at A and D, respectively.
4. The North Pacific subtropical gyre is downwind of the China seas (Figure 1). It is not surprising that dust particles from B and C–D–E–F affected both areas due to surface-level northwesterly winds induced by the Asian winter monsoon and/or by westerly winds in the free troposphere from the eastern Asian continent to the Pacific Ocean [28]. The North Pacific subtropical gyre is not light limited [15] and is instead P limited or Fe–P co-limited [12]. Much dust (Table S1) reached the sea in spring (75%), summer (13%), and winter (7%). In addition, the total dust deposition also showed high deposition in spring and summer (Table 3). Thus, dust could provide a winter and springtime nutrient inventory and could contribute to the summer bloom in this region, according to the time of aerosol Fe dissolution and the lag time of 3 weeks to 1 month of bloom relative to dust deposition [7], [12]. Uematsu *et al*., [29] reported that the annual deposition flux decreased rapidly from the coastal area (21 g m^−2^ a^−1^) to the open ocean (0.8 g m^−2^ a^−1^) over the western North Pacific. The GMOD simulated total dust deposition also showed that the deposition in the China seas was larger than that in the North Pacific subtropical gyre because of transport decay (Table 3 and Figure 4). This made the correlation coefficients of B and C–D–E–F with the China seas higher than those with the North Pacific subtropical gyre (Figure 3D).
5. Based on the correlations obtained, the dust particles in C–D–E–F could affect chlorophyll *a* concentrations in the western Equatorial Pacific and the eastern Equatorial Pacific (Figure 3E and 3F). Longhurst, [15] found that these two areas were limited by nutrient or trace elements but not by light. However, very few dust storms can arrive in these two ocean regions (Table 2) and the annual total dust deposition is smaller than 11 g m^−2^ a^−1^ (Table 3 and Figure 4). The lowest correlation was thus observed for a long-term period. Additionally, the most important source of Fe for the eastern Equatorial Pacific surface waters is the water from the Equatorial Undercurrent [7], [30].

**Table 3:** Simulated total dust deposition (including dry and wet depositions, unit: g m^−2^ a^−1^).

Sea areas	spring	summer	autumn	winter	annual
China seas	16	16	8	6	46
Sea of Japan	23	13	9	5	50
subarctic North Pacific	5	5	3	1	14
North Pacific subtropical gyre	14	15	7	4	40
western Equatorial Pacific	2	3	3	3	11
eastern Equatorial Pacific	2	3	1	1	7

The correlations for each pair of sea area and dust source could also result from reasons other than those discussed above. The possibility appeared to be low when the correlation between chlorophyll *a* concentration in the coastal region (<50 m) and dust storms (Figure S4) was analyzed as follows: 1) No significant correlation existed between dust storm and chlorophyll *a* concentration in the coastal region of the China seas because of the considerable impact of large rivers (the Yangtze and Yellow River). Table 1 also shows that nitrate and phosphate concentrations in the coastal region of the China seas were about two times higher than those in remote regions (>50 m). The same is true for the coastal sea of the equatorial Pacific (Figure S4). 2) In the coastal region of the Sea of Japan, the North Pacific subtropical gyre and the subarctic North Pacific, correlation between the chlorophyll *a* concentration and dust storms were observed. This was due to the fact that nutrient conditions in the coastal regions of the three seas were similar to those in remote regions (Table 1) because of the low river discharge [31].

### Conclusions

During 1997–2007, several centers with high occurrence frequency of Asian dust storms were identified in China: the Taklimakan Desert, the west of Inner Mongolia, the Hunshandake Desert, the Qaidam Basin and Kumtag Deserts, the Hexi Corridor, the Hetao Region, and the Horqin Sandy Land in descending order. The occurrence frequency of dust storms in the Hunshandake Desert increased in 1997–2007 relative to that in 1954–1996. The reverse was true in the other centers where the same comparison was conducted.

The correlation obtained between the frequencies of dust storms reaching each sea and chlorophyll *a* concentration in each sea suggested that Asian dust could provide nutrients to the seas and promote phytoplankton growth, even in the marginal seas. Among these seas, the correlation for the Sea of Japan was the highest except that the correlation of the subarctic North Pacific with the Taklimakan Desert was higher, followed by the China seas and the North Pacific subtropical gyre. Although Asian dust rarely reached the western and eastern Equatorial Pacific, the correlation observed between dust storm frequencies and their chlorophyll *a* concentrations suggested that Asian dust could affect their biological activities.

Although phytoplankton growth in the China seas was expected to be more sensitive to external nutrient inputs than the Sea of Japan due to lower macronutrient concentrations, the correlation between dust storms and chlorophyll *a* concentration in the former was lower than that in the latter. This may be due to the fact that more dust storms could reach and deposit into the Sea of Japan. Additionally, it seems that the impact of toxicity from anthropogenic metals associated with Asian dust could be present in the China seas. This needs further investigation.

## Supporting Information

Figure S1 **The forward trajectories from dust source zone G (Tongyu station) at 3 km (A) and 7 km (B).**
(TIF)

Figure S2 **The pathways of Asian dust storms to the sea from Zhang and Gao,** [**21**] **which was added over annual averaged MODIS aerosol optical depth.**
(EPS)

Figure S3 **The annual averaged Ozone Monitoring Instrument NO_2_ column densities.**
(EPS)

Figure S4 **The correlation between monthly chlorophyll** ***a*** **concentration (mg m^−3^) in the six sea areas with a depth shallower than 50 m and monthly occurrence frequency of dust storms (days per month) for the period from September 1997 to December 2007.** The minimum contour is 0.2 with significance level of 0.05. The stations with the largest correlation coefficient were shown. A1, A2, A3, B1, B3, D2, F1, F3, G1, and G3 are the stations of Minfeng, Alar, Yarkand, Mangya, Xiaozaohuo, Mazongshan, Sunitezuoqi, Narenbaolige, Tongyu and Fuxin, respectively.
(TIF)

Table S1 **The seasonal ratio (percentage) of dust storms reaching the six study sea areas based on forward trajectories.**
(PDF)

## References

[1] Zhang X-Y, Gong S-L, Zhao T-L, Arimoto R (2003) Sources of Asian dust and role of climate change versus desertification in Asian dust emission. Geophysical Research Letters 30: 2272 doi:2210.1029/2003GL018206.

[2] Duce RA, LaRoche J, Altieri K, Arrigo KR, Baker AR, et al (2008) Impacts of Atmospheric Anthropogenic Nitrogen on the Open Ocean. Science 320: 893–897. 18487184 10.1126/science.1150369

[3] Jickells TD, An ZS, Andersen KK, Baker AR, Bergametti G, et al (2005) Global iron connections between desert dust, ocean biogeochemistry, and climate. Science 308: 67–71. 15802595 10.1126/science.1105959

[4] Furutani H, Meguro A, Iguchi H, Uematsu M (2010) Geographical distribution and sources of phosphorus in atmospheric aerosol over the North Pacific Ocean. Geophysical Research Letters 37: L03805 doi:03810.01029/02009GL041367.

[5] Shi J, Gao H, Qi J, Zhang J, Yao X (2010) Sources, compositions, and distributions of water-soluble organic nitrogen in aerosols over the China Sea. Journal of Geophysical Research 115: D17303 doi:17310.11029/12009JD013238.

[6] Baker AR, Croot PL (2010) Atmospheric and marine controls on aerosol iron solubility in seawater. Marine Chemistry 120: 4–13.

[7] Boyd PW, Mackie DS, Hunter KA (2010) Aerosol iron deposition to the surface ocean - Modes of iron supply and biological responses. Marine Chemistry 120: 128–143.

[8] Chan CK, Yao X (2008) Air pollution in mega cities in China. Atmospheric Environment 42: 1–42.

[9] Bishop JKB, Davis RE, Sherman JT (2002) Robotic observations of dust storm enhancement of carbon biomass in the North Pacific. Science 298: 817–821. 12399588 10.1126/science.1074961

[10] Yuan W, Zhang J (2006) High correlations between Asian dust events and biological productivity in the western North Pacific. Geophysical Research Letters 33: L07603 doi:07610.01029/02005GL025174.

[11] Jo CO, Lee J-Y, Park K-A, Kim YH, Kim K-R (2007) Asian dust initiated early spring bloom in the northern East/Japan Sea. Geophysical Research Letters 34: L05602 doi:05610.01029/02006GL027395.

[12] Calil PHR, Doney SC, Yumimoto K, Eguchi K, Takemura T (2011) Episodic upwelling and dust deposition as bloom triggers in low-nutrient, low-chlorophyll regions. Journal of Geophysical Research 116: C06030 doi:06010.01029/02010JC006704.

[13] Tan S-C, Shi G-Y, Shi J-H, Gao H-W, Yao X (2011) Correlation of Asian dust with chlorophyll and primary productivity in the coastal seas of China during the period from 1998 to 2008. Journal of Geophysical Research 116: G02029 doi:02010.01029/02010JG001456.

[14] Han Y, Zhao T, Song L, Fang X, Yin Y, et al (2011) A linkage between Asian dust, dissolved iron and marine export production in the deep ocean. Atmospheric Environment 45: 4291–4298.

[15] Longhurst A (1995) Seasonal cycles of pelagic production and consumption. Progress In Oceanography 36: 77–167.

[16] Qian Z-A, Cai Y, Liu J-T, Liu C-M, Li D-L, et al (2006) Some advances in dust storm research over China-Mongolia areas (in Chinese). Chinese Journal of Geophysics 49: 83–92.

[17] Draxler RR, Rolph GD (2010) HYSPLIT (HYbrid Single-Particle Lagrangian Integrated Trajectory) Model access via NOAA ARL READY Website (http://ready.arl.noaa.gov/HYSPLIT.php).NOAA Air Resources Laboratory, Silver Spring, MD.

[18] Mikami M, Shi GY, Uno I, Yabuki S, Iwasaka Y, et al (2006) Aeolian dust experiment on climate impact: An overview of Japan-China joint project ADEC. Global and Planetary Change 52: 142–172.

[19] Matsuki A, Iwasaka Y, Osada K, Matsunaga K, Kido M, et al (2003) Seasonal dependence of the long-range transport and vertical distribution of free tropospheric aerosols over east Asia: On the basis of aircraft and lidar measurements and isentropic trajectory analysis. Journal of Geophysical Research 108: 8663–8676 doi:8610.1029/2002JD003266.

[20] Yue X, Wang H, Wang Z, Fan K (2009) Simulation of dust aerosol radiative feedback using the Global Transport Model of Dust: 1. Dust cycle and validation. Journal of Geophysical Research 114: D10202.

[21] Zhang K, Gao H-W (2007) The characteristics of Asian-dust storms during 2000–2002: From the source to the sea. Atmospheric Environment 41: 9136–9145.

[22] Liu XH, Ding RQ (2007) The relationship between the Spring Asian Atmospheric circulation and the previous winter Northern Hemisphere annular mode. Theoretical and Applied Climatology 88: 71–81.

[23] Wang B-D, Wang X-L, Zhan R (2003) Nutrient conditions in the Yellow Sea and the East China Sea. Estuarine, Coastal and Shelf Science 58: 127–136.

[24] Wu J, Chung SW, Wen LS, Liu KK, Chen YL, et al (2003) Dissolved inorganic phosphorus, dissolved iron, and Trichodesmium in the oligotrophic South China Sea. Global Biogeochemical Cycles 17: 1008–1017.

[25] Tang Y, Carmichael GR, Kurata G, Uno I, Weber RJ, et al (2004) Impacts of dust on regional tropospheric chemistry during the ACE-Asia experiment: A model study with observations. Journal of Geophysical Research 109: D19S21 doi:10.1029/2003jd003806.

[26] Takata H, Kuma K, Isoda Y, Otosaka S, Senjyu T, et al (2008) Iron in the Japan Sea and its implications for the physical processes in deep water. Geophysical Research Letters 35: L02606 doi: 02610.01029/02007gl031794.

[27] Paytan A, Mackey KRM, Chen Y, Lima ID, Doney SC, et al (2009) Toxicity of atmospheric aerosols on marine phytoplankton. Proceedings of the National Academy of Sciences of the United States of America 106: 4601–4605. 19273845 10.1073/pnas.0811486106 PMC2653564

[28] Zhao TL, Gong SL, Zhang XY, Blanchet J-P, McKendry IG, et al (2006) A Simulated Climatology of Asian Dust Aerosol and Its Trans-Pacific Transport. Part I: Mean Climate and Validation. Journal of Climate 19: 88–103.

[29] Uematsu M, Wang Z, Uno I (2003) Atmospheric input of mineral dust to the western North Pacific region based on direct measurements and a regional chemical transport model. Geophysical Research Letters 30: 1342 doi:1310.1029/2002GL016645.

[30] Coale KH, Fitzwater SE, Gordon RM, Johnson KS, Barber RT (1996) Control of community growth and export production by upwelled iron in the equatorial Pacific Ocean. Nature 379: 621–624.

[31] Nohara D, Kitoh A, Hosaka M, Oki T (2006) Impact of Climate Change on River Discharge Projected by Multimodel Ensemble. Journal of Hydrometeorology 7: 1076–1089.

