# Supplementary figures and images for: Variability in the Correlation between Asian Dust Storms and Chlorophyll a Concentration from the North to Equatorial Pacific
Source: PLoS One. 2013 Feb 27;8(2):e57656. doi: 10.1371/journal.pone.0057656 (PMC3584023; doi:10.1371/journal.pone.0057656)

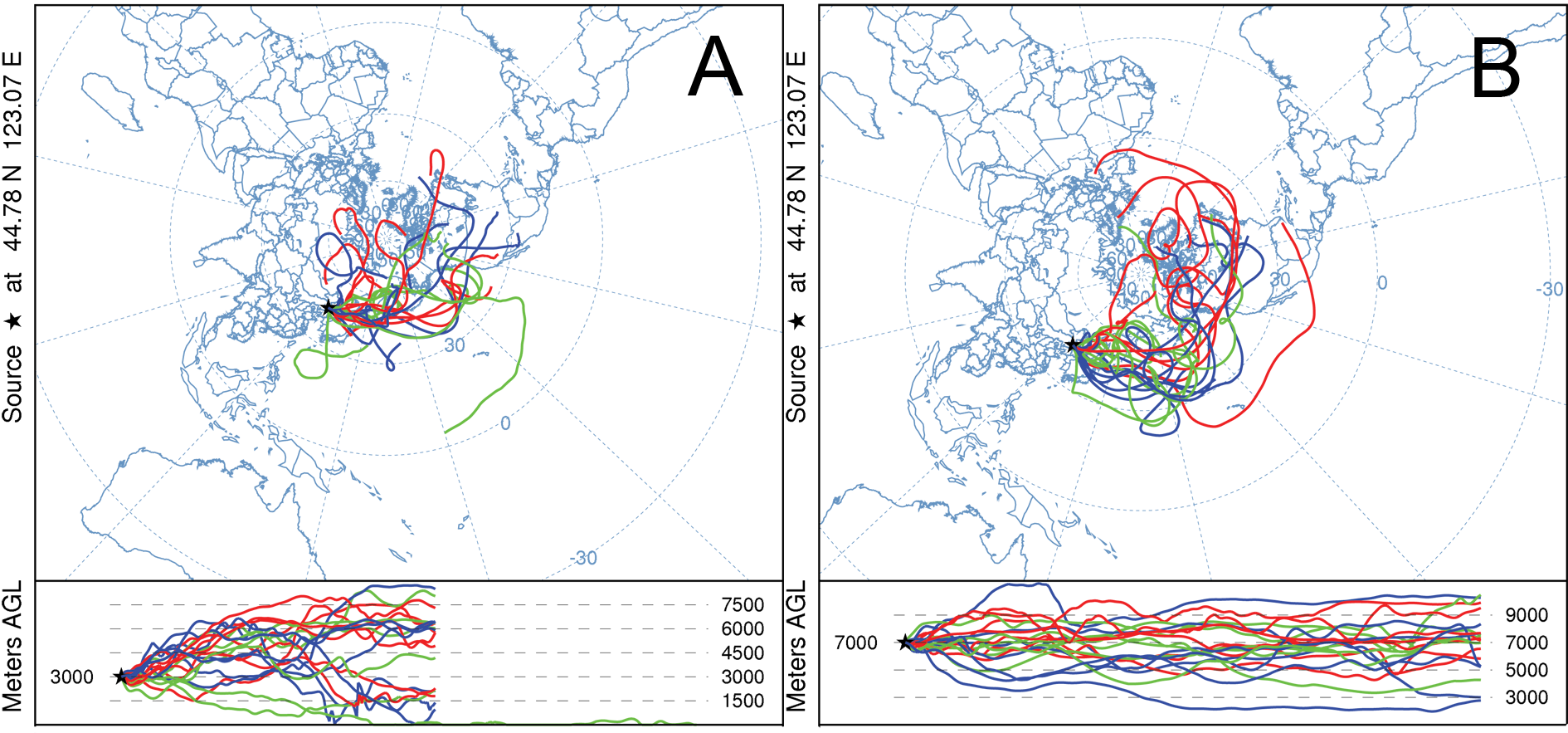

Supplement: Figure S1 — The forward trajectories from dust source zone G (Tongyu station) at 3 km (A) and 7 km (B). (TIF) [file pone.0057656.s001.tif]

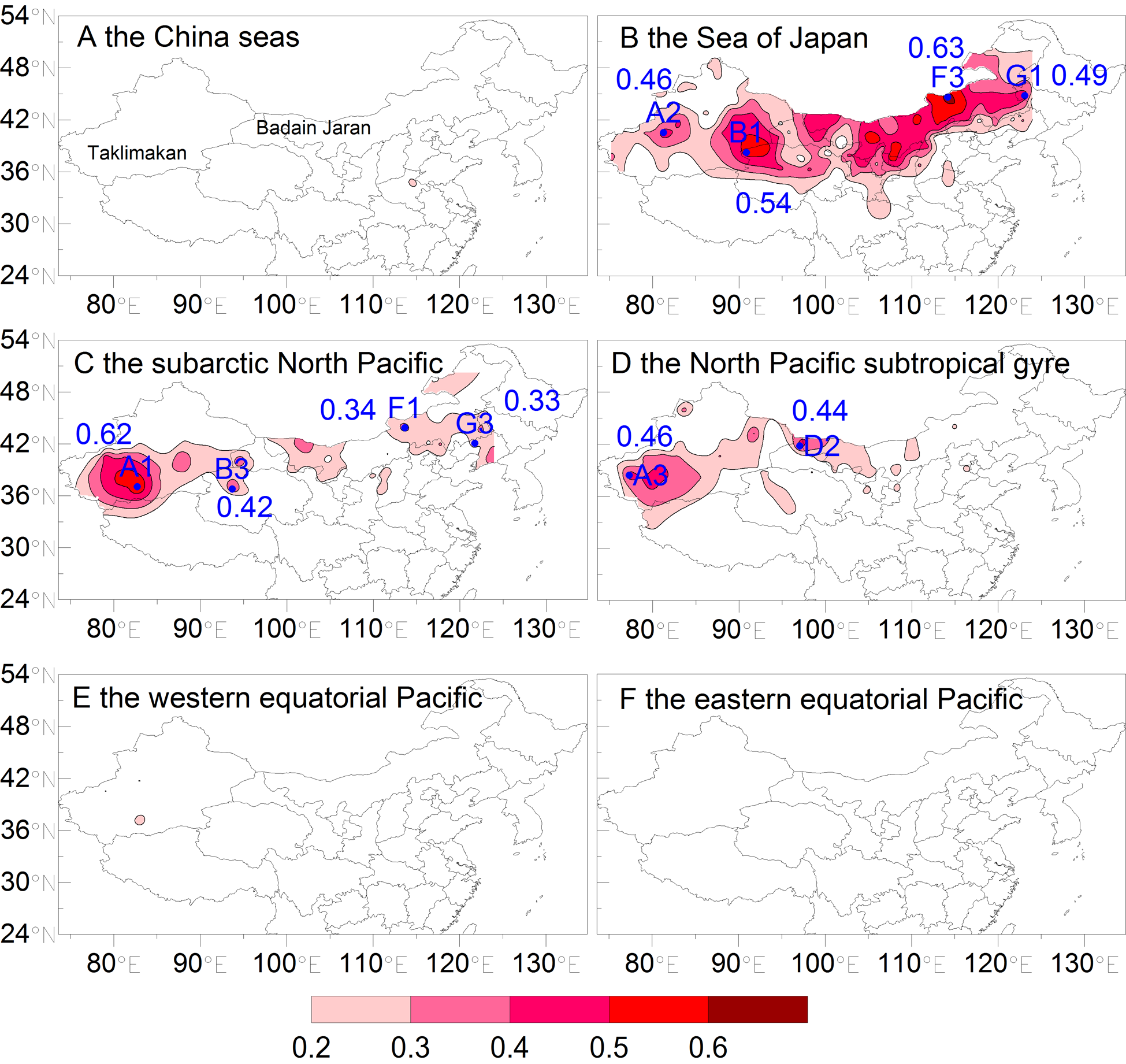

Supplement: Figure S4 — The correlation between monthly chlorophyll a concentration (mg m−3) in the six sea areas with a depth shallower than 50 m and monthly occurrence frequency of dust storms (days per month) for the period from September 1997 to December 2007. The minimum contour is 0.2 with significance level of 0.05. The stations with the largest correlation coefficient were shown. A1, A2, A3, B1, B3, D2, F1, F3, G1, and G3 are the stations of Minfeng, Alar, Yarkand, Mangya, Xiaozaohuo, Mazongshan, Sunitezuoqi, Narenbaolige, Tongyu and Fuxin, respectively. (TIF) [file pone.0057656.s004.tif]
