# Supplementary material for: Variability in the Correlation between Asian Dust Storms and Chlorophyll a Concentration from the North to Equatorial Pacific
Source: PLoS One. 2013 Feb 27;8(2):e57656. doi: 10.1371/journal.pone.0057656 (PMC3584023; doi:10.1371/journal.pone.0057656)
Supplement: Table S1 — The seasonal ratio (percentage) of dust storms reaching the six study sea areas based on forward trajectories. (PDF) [file pone.0057656.s005.pdf]

Table S1. The seasonal ratio (percentage) of dust storms reaching the six study sea areas based on forward trajectories.

Season	China seas	Sea of Japan	subarctic North Pacific	North Pacific subtropical gyre	western Equatorial Pacific	eastern Equatorial Pacific
Spring	68	66	69	75	73	81
Summer	15	14	12	13	20	13
Fall	8	11	11	5	4	4
winter	9	9	8	7	3	2
